# Mobile Game Evaluation Method Based on Data Mining of Affective Time Series

**DOI:** 10.3390/s25092756

**Published:** 2025-04-26

**Authors:** Jeremi K. Ochab, Paweł Węgrzyn, Przemek Witaszczyk, Dominika Drążyk, Grzegorz J. Nalepa

**Affiliations:** 1 Institute of Theoretical Physics, Mark Kac Complex Systems Research Centre, Jagiellonian University, 30-348 Kraków, Poland; jeremi.ochab@uj.edu.pl; 2 Jagiellonian Human-Centered Artificial Intelligence Laboratory (JAHCAI), Institute of Applied Computer Science, 30-348 Kraków, Poland; pawel.wegrzyn@uj.edu.pl; 3 Institute of Philosophy, Jagiellonian University, 31-007 Kraków, Poland; dominika.a.drazyk@gmail.com

**Keywords:** game data mining, affective computing, mobile video games

## Abstract

Our work is positioned at the intersection of game data science, affective gaming, and the implementation of multimodal body sensors analysis. We propose an original method of evaluating the quality of a class of video games based on the emotional reactions of players. Game developers ask why some games are more profitable (MP games) than others (LP games). An intuitively convincing hypothesis is often put forward: MP games evoke more positive emotions and hence are sustainably engaging. Our main hypothesis is that test players who can clearly distinguish between MP game and LP game in relatively short test sessions are more reliable in scoring games and valuable to keep track of their emotions. From a random group of test players, we selected players with such abilities. We analyzed their affective spectra and obtained a fairly clear confirmation that the selected players showed more positive and less negative emotions in MP games than in LP ones. We can reasonably expect these players to be focused on playing in the test session, and their emotions may really indicate the strengths of MP games over LP games. We present the results of the experimental evaluation of our method conducted with with a leading game company in Poland.

## 1. Introduction

Video games are an interesting cultural phenomenon which has become an important part of the global industry. As games and artificial intelligence (AI) have always been interrelated, there has been many works in the area of the applications of the most recent AI methods in video games. In the last decades, AI techniques have been used in games to drive and augment the game logic and mechanics, e.g., the control of NPCs (Non-Playable Characters). Furthermore, there has been a growing interest in the use of AI for game design, e.g., for content generation, graphics effacement, and so forth [1].

However, an even more emerging trend is that games are also becoming a new area of study for computational methods and data science. In fact, they provide vast amounts of behavioral data that can allow for a better understanding of players’ actions, choices, preferences, and even emotions. Today, game engines are able to log not only game events and player interactions but also external behavioral and physiological data from the interfaces such as keyboards and gamepads. This spectrum can be further extended in laboratory environments with additional devices. This research direction has scientific and commercial potential, as it allows for better adaptation and personalization of games, as well as also for insights regarding the perception of games by players. This rapidly growing approach has been called game data mining [2] or game data science [3].

One of the areas in gaming that has attracted a lot of attention is affective gaming. The idea of the development and application of affective computing (AfC) [4] techniques in games was proposed over a decade ago [5]. It regards several perspectives regarding emotions in games, including the design of affective games, detection and prediction of player emotions during games, and emotion elicitation via games [6]. This approach clearly overlaps with the concept of game data science and uses its techniques.

Our work is positioned at the intersection of game data science and affective gaming. In this paper, we propose an original method to evaluate the quality of a selected class of video games based on the emotional reactions of players.

Our main hypothesis is that test players who can clearly distinguish between already published more profitable (MP) and less profitable (LP) games in relatively short-test gameplay sessions are more reliable in scoring games and valuable to keep track of their emotions. This idea is closely related to the aforementioned intuitive mechanism that games inducing more positive emotions lead to enduring engagement of the player and, hence, monetization potential. Properly estimating these emotions (i.e., attributing them reliably to game properties and not some unrelated factors) is a challenge in this approach, and our research aims at providing a robust mechanism for selecting naturally reliable testers.

We focus on mobile games, as the market for them has been most rapidly growing in recent years (Revenue in the mobile games segment is projected to reach 124,905 m. USD in 2022. Revenue is expected to show an annual growth rate (CAGR 2022–2026) of 8.73%, resulting in a projected market volume of 174,586 m. USD by 2026. In the mobile games segment, the number of users is expected to amount to 2309.4 m. users by 2026. The average revenue per user in the mobile games segment is projected to amount to 64.66 USD in 2022; see https://www.statista.com/outlook/dmo/digital-media/video-games/mobile-games/worldwide (accessed on 14 February 2025)). Furthermore, mobile phones offer a range of additional sensors, providing a much richer spectrum of data for analysis. Moreover, the market for mobile games distributed solely via dedicated online stores such as Google Play has pioneered new business models for generating income from games. In our method, we consider a large class of free-to-play (F2P) games that offer additional in-app purchases (IAPs). The “quality” we consider is in fact directly related to the potential for monetization and commercial success in F2P games. We introduced an experimental workflow to select a proper group of players that evaluate the given F2P games. We then recorded their emotional reactions during game play. Based on these data, as well as additional knowledge about player characteristics and domain knowledge regarding the game design, we predicted whether the given games could be successful from a commercial perspective. Finally, we present and discuss the results of the pilot evaluation of our method, which was conducted in close cooperation with one of the leading game companies in Poland.

The rest of the paper is structured as follows. In Section 2, we introduce our method. The setup of the practical evaluation, including the data we gathered, is described in detail in Section 3. We describe the analysis of these data as well as the evaluation of our method in Section 4. We summarize the paper and propose directions for future works in Section 5.

## 2. Novel Approach to Gameplay Evaluation

### 2.1. Motivation

The objective of our method is to assess the quality of mobile games based on the emotional responses of individual players testing them as an objective measure of the players’ underlying engagement. We focus specifically on free-to-play or “freemium” (“free” and “premium”) mobile games that give players access to their content without paying. (The global free-to-play mobile games market was estimated at 73.8 billion USD in 2020, and this figure is predicted to rise to 75.6 billion USD by 2021. https://www.statista.com/statistics/1107021/f2p-mobile-games-revenue/ (accessed on 14 February 2025)). The developer makes profit from players who start to play these games and will be willing to reach for them often. This is possible when players exhibit a strong commitment, which we stipulate to be linked to a positive emotional attitude toward these games. Hence, developers try to analyze emotions during trial tests on players that are performed during the process of game design and production. However, an operational understanding of “game quality” is needed to perform the evaluation. For game players, the positive reception of the game in the first days and weeks of game playing is decisive for further interest and engagement.

Our approach is based on combining questionnaire interviews with game testers while tracking naturalistic signs of emotions. Surveys are valuable, but they have their limitations (see [7,8]).

First, the game tester may intentionally or unconsciously hide their true feelings about the game, introducing bias into the data. Second, we obtain from the game tester a rating of the whole game, not its individual elements, and we would like to aim at devising a finer-grained tool.

AfC methods produce data that allow us to analyze the player’s emotions more objectively and at different times.

The biggest challenge in detecting emotions based on naturalistic data (speech, facial expression, body gesture, physiological monitoring) is to train the classifier so that it is universal for different people. There is a large body of work dedicated to various approaches to this problem. Selected examples are as follows. In multimodal settings, one can analyze, e.g., heart rate, skin conductivity, or audiovisual data [9,10,11,12,13]. Facial expressions central to our approach were studied in, e.g., [14]. Psychological aspects of emotion processing were pursued in [15]. Classifier construction has been the focus of research in, e.g., [16,17]. The idea of arousal valence, which we employ in our study, has also been explored in [18].

However, even after having a proper method for recognizing player emotion (which often is a challenge in itself), one is still unsure as to whether the identified emotions of the player really relate to the game. Moreover, the first few levels of any game are usually not enough for most of the respondents to experience deep immersion and strong emotions associated with this state. Furthermore, the game tester may have a good day, or a particular game genre has elicited his liking and engaged him in the game.

### 2.2. Assessment Method

**Figure 1:**
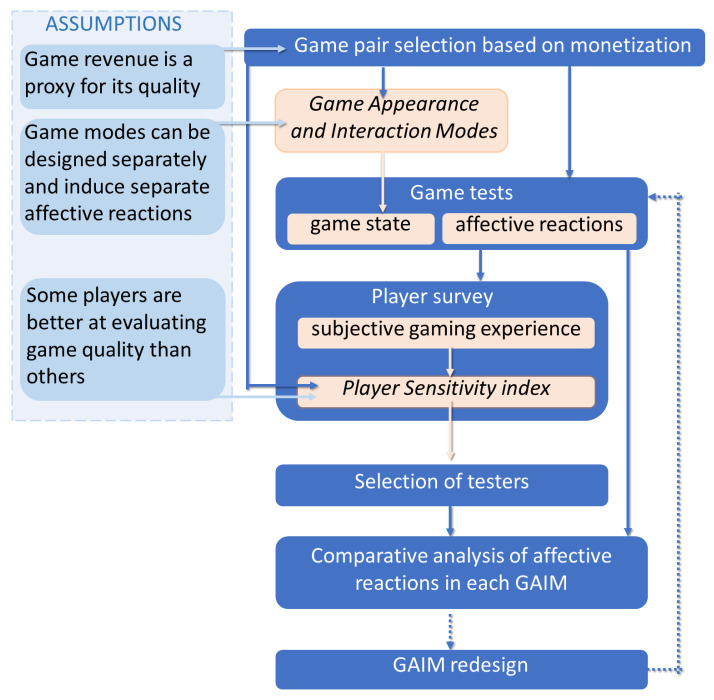
Flow chart of the approach to game evaluation. The crucial conceptual steps are to divide the game into Game Appearance and Interaction Modes (GAIMs) and to select the players who can distinguish between the more and less profitable games (MP and LP). Next, after data acquisition, affective data mining begins. At this stage of our study, we did not perform the UX redesign loop (dotted arrows).

To address these challenges, we developed an original method for the assessment of quality of mobile F2P games. The general flow chart is presented in Figure 1. Its main steps are as follows:

1. Introducing a specific understanding of quality based on the game’s financial success recorded drawn from independent sources. See Section 2.3.
2. Selecting pairs of games of specific type or genre, with one of the games having good and the other poor quality. See Section 3.1.
3. Performing a series of game tests in a laboratory environment with a possibly broad group of testers. See Section 3.3 and Section 3.4.
4. Recording selected affective reactions of the testers for further analysis. See Section 2.6 and Section 3.5.
5. Players reporting experiences in an evaluation survey after each game session. See Section 3.6.
6. Introducing the concept of player sensitivity index based on responses to specific questions from the survey. See Section 3.7.
7. Ranking testers based on the sensitivity index and selecting the ones whose responses are the most credible. See Section 3.8.
8. Narrowing down the analysis of affective recordings to the above testers.
9. Finally, introducing the concept of specific game modules, which allows us to interpret the results of the analysis in a given context of the mobile game. See Section 2.5 and Section 3.2.

The idea of combining quantitative (direct measurements, e.g., physiological) and qualitative data (interviews, observations, and questionnaires) in the analysis of players’ emotions is not new. For instance, in [19], Granato analyzed the relation between the physiological data and the self-assessment provided by the player. We take a different approach, wherein subjective player ratings are not used to verify or interpret players’ emotional states. Instead, they are used to assess whether a player can distinguish between an MP and an LP game. Only then do we quantify the emotions of such select players and search for differences between the game pair. These two steps are independent in terms of the data used.

Below, we describe the founding concepts of the method, which were jointly developed by the research team with the experts from the gaming company we worked with.

### 2.3. Assessment of Game Quality

Based of the business perspective of the game developers, the main factor that constitutes an F2P game as a “good one” is directly related to its financial success, i.e., monetization. For simplicity, we want to be able to distinguish between highly and poorly monetizing games (which hereafter we call *more profitable*, MP, and *less profitable*, LP, games for brevity). The data regarding monetization are accessible to game developers through dedicated portals. The experts from the company selected three specific factors: (1) application score in the app store (S), (2) the number of downloads (D), and (3) the total income from the game (I). In this context, MP games are ones being in the top 10% for all the three factors S, D, I, whereas LP games are ones in the lowest 10% for all of them.

### 2.4. Player Sensitivity Index

We propose two specific indices quantifying a player’s ability to correctly distinguish MP from LP games. They were designed to express, broadly speaking, the player’s sensitivity to the game’s user experience (UX) and, specifically, the player’s ability to experience intense positive (negative) emotions when playing a better (worse)-designed UX. The indices are based on responses to the after-game evaluation survey (see Data Availability section) about a person’s interest in the game and their experience of playing the game. The player’s *base index* is based on questions directly related to monetization, expressing readiness to spend money in/for the game, playing it in the future, and recommending it. The player’s *UX sensitivity index* is based on questions related to theemotional response to the game, e.g., if it was interesting, exciting, fun, relaxing, or enjoyable.

### 2.5. GAIMs

We introduce the concept of GAIM (Game Appearance and Interactive Mode), which defines a game module of universal function, regardless of the particular game example. From the game designer’s perspective, it is instantiated in the game code as a separate part of the application logic and a separate view within the user interface. From the player’s point of view, it is a different type of game screen and a form of interaction (e.g., tutorial, main gameplay, character screen, inventory screen, summary screen, or victory screen).

Such a concept is needed, since instead of just evaluating the game as a whole, we would like to provide UI and UX designers with a method that helps to evaluate particular game elements. The need for finer-grained modes is based on the following assumptions:

1. Each type of interaction with the game interface can individually induce a separate affective reaction.
2. The optimal composition of affective reactions that designers should aim for can be different for each mode.

GAIMs, understood in this way, can be treated as separate units for both UI and UX design, as well as for conducting affective research on player reactions. We are not aware of other game user research that has operationalized a similar concept.

The GAIM’s definition is functional. As such, it allows for exceptions to the separation criterion: The prime example is the tutorial, which typically should overlap with most of the other GAIMs in order to provide the player with a comprehensive introduction to all the elements of the gameplay. Due to the functional definition, GAIMs can be designed and implemented differently in different games. The repertoire of GAIMs may also differ, although we assume that it would typically strongly overlap within games of the same type. This assumption is important for the reliability of comparative analyses like ours.

### 2.6. Affective Reactions

During the experiment, we aimed at recording a range of affective signals. Based on our previous experience [20] for this work, we decided to select a single source of affective signals regarding face expressions. This method is based on the classic research by P. Ekman [21]. In game user research, facial expressions were often recorded via facial electromyography (see, e.g., [22]), which brought additional limitations. Currently, facial expression recognition from 2D video recording is one of the most accessible state-of-the-art methods implemented in a number of computer tools (see, e.g., [23], and the survey [24] or [25] for discussion of some limiations).

## 3. Materials and Methods

To practically evaluate our method, we prepared and performed a pilot study in a laboratory environment provided by the partner video games company.

### 3.1. Selected Games

Three F2P mobile game types were studied that represent three dominant game mechanics types in the freemium sector, with one pair of MP–LP games each. The particular games were selected by an in-house game design expert from among the games that jointly met conditions based on the three factors listed in Section 2.3, i.e, revenue, downloads, and user scores. MP games were chosen from among those that were in the top 10% for all three factors, while the LP games were chosen from the bottom 10% for all three factors. Data on the above factors were obtained from the https://appmagic.rocks (accessed on 14 February 2025) portal. The selection used in our research is as follows:

Game type: Match-3. Titles: Animatch (MP), Clockmaker (LP);
Game type: Card Strategy. Titles: Legendary (MP), Match Land (LP);
Game type: Card RPG. Titles: Clash Royale (MP), Card Heroes (LP).

We shall refer to the particular games as m3-MP, m3-LP, cStr-MP, cStr-LP, and *cRPG-MP*, *cRPG-LP* for brievity. The m3-MP game was developed by the company that conducted the project.

### 3.2. GAIM Partitioning

The time course of the gameplay was partitioned into the following GAIMs: *gameplay*—the main gameplay; *navigation*—the main navigation screen; *won*—summary of a game won; *lost*—summary of a game lost; *collection*—interaction with card collection screen; *hero*—interaction with the player’s character’s screen; *shop*—interaction with the shop screen; *tutorial*—introduction to the chosen (or all) elements of gameplay; *ad*—in-app advertisements, if present; and *none*—where no other GAIM was tagged. The GAIMs available for analysis differed depending on game type, as listed in Table 1. The identification and timing of GAIMs, as well as any interruptions in data acquisition due to technical problems, were recorded manually. See Section 3.4 for details about such interruptions.

### 3.3. Participants

The volunteers were remunerated for participation in the experiment, and they signed a consent form. They were informed that the aim of the measurements was testing players’ reactions to certain gameplay elements. There were 68 participants (31 female, 37 male) aged 24.8±4.6 y.o. They were enrolled for the study based on the recruitment questionnaire, which is available in Data Availability section. The inclusion criteria was 18 y.o. or above and playing mobile games. There were 87% of participants that declared playing match-3 games, 74% playing mobile RPG games, and 56% who considered themselves active members of the gaming community. There was one person declaring playing more than 20 h/week, 22% playing 11–22 h/week, 56% playing 3–10 h/week, and 21% playing less than 2h/week. In terms of Bartle’s taxonomy of player types [26], there were 46% explorers, 28% achievers, 21% killers, and 6% socializers.

### 3.4. Experimental Procedure

**Figure 2:**
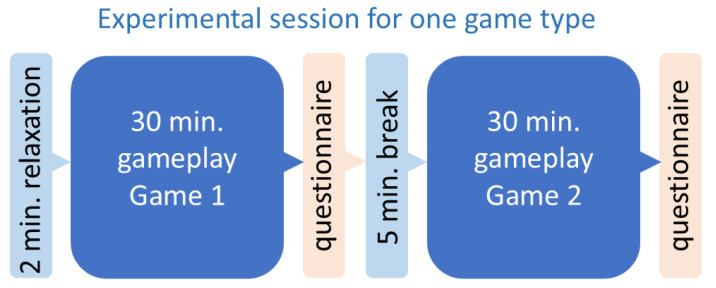
Experimental session for a pair of games of a given type. The order of MP and LP games was randomized.

The structure of the experimental session is shown in Figure 2. Each subject participated in three 1-h gameplay blocks (30-min session for each of the six games, preceded by a 2-min relaxation period). The two games in a given block were matched according to the type. The order of the games (MP first, LP second, or vice versa) was randomized. After each session, the participants filled out an evaluation survey measuring interest in the game; there were 5-min breaks between the sessions. The gameplay was performed on smartphones and with headphones provided by the experimenters. The game data were reset for each participant, even if they had played the game before. The smartphone’s screen was recorded throughout the whole gameplay and so was, by a separate desktop webcam, the participant’s face. Additionally, heart rate (HR) and electrodermal activity (EDA) were recorded during each session. Here, we do not report any results on HR/EDA.

The experimental procedure was conducted by the company’s staff trained by the authors. The room temperature was set to 23 °C, and the blinds in the rooms were lowered. There were up to two participants in the room simultaneously, separated by desktop office screens.

In total, there were 23 interruptions (11% of sessions) of various kinds and three cases of the state of the game not being reset. These interruptions included events affecting user experience (game advertisement, game crashing, game updating, screen freezing, disconnecting the sound, accidental screen locking or opening a phone’s functionality, or a notification showing up on top of the game screen) and fire alarm in the building. Additionally, the time series was tagged for issues that might have affected facial expression recognition (participant’s face out of camera frame, participant’s face covered, participant touching their head or headphones, webcam not recording due to a software crash) and HR/EDA time series (participant touching the electrodes). All the incidents were tagged in the analyzed gameplay data.

### 3.5. Affective Time Series

**Figure 3:**
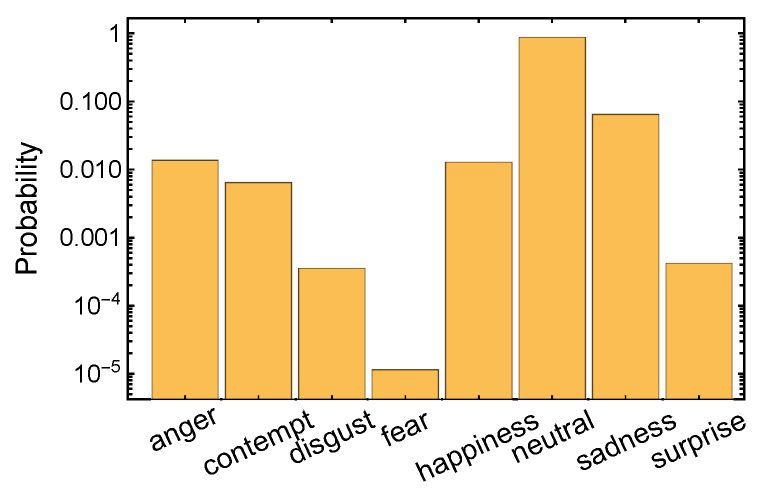
Histogram of the emotions recognized from facial expressions collected over all participants during the whole gameplay in all six games. Note the logarithmic scale and the dominance of neutral emotion.

**Figure 4:**
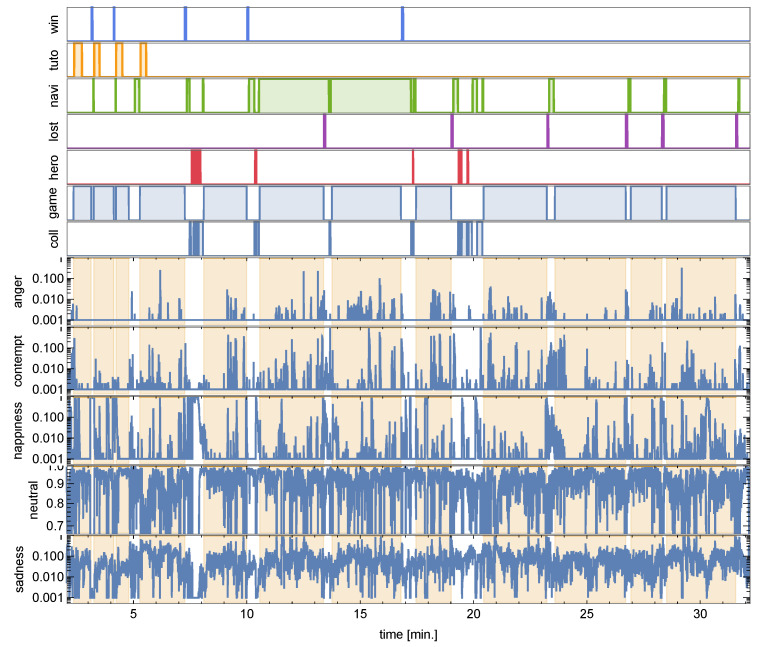
Affective time series of a single player (participant 47) of cStr-MP. Top rows indicate occurrence of each GAIM. Bottom rows are log-scaled probabilities of emotions correlatedwith GAIM changes. The yellow-shaded areas indicate *gameplay*. Note the drop in *sadness* and peak in *happiness* in the first *hero* GAIM and similar changes during *tutorial*.

By affective time series (ATS), we generally mean temporally annotated information useful in analyzing affective states of players (gameplay recordings, game logs, physiological signals). In the experiment, ATS comprised emotions inferred from facial expressions of the participants complemented by the timing of GAIMs. The facial expression recognition was performed with an off-the-shelf solution by Microsoft Azure Face service (See https://docs.microsoft.com/en-us/azure/cognitive-services/face/ (accessed on 14 February 2025)). The frame rate of facial recordings was downsampled to 4 fps for that purpose. We focused on nine attributes provided by the classifier: *smile* (returned by the Microsoft Azure system as a float in the range [0, 1]) and eight emotional states—*anger*, *contempt*, *disgust*, *fear*, *happiness*, *neutral*, *sadness*, *surprise*. The numeric values representing confidence of occurrence of each emotion were normalized to the range [0,1], summing up to 1 at each time step. Typically, at each time point, several emotions were present, although differing by up to 3 orders of magnitude cf. Figure 3 shows the totals, and Figure 4 shows their temporal trajectories. Notably, the most prevalent the emotion was *neutral*. This can be partly attributed to the physical limitations of the mobile device. Being much smaller than a computer screen and hence requiring much more focused stance, the human faces seemed more neutral to the classifier than the actual gamers’ engagement would indicate. Another reason is that the analyzed games were less action-intensive than full-scale competitive PC games, since our focus group was more player–game and relation–development-driven than action-driven.

### 3.6. After-Game Evaluation Survey

The after-game evaluation survey is available in Data Availability section. The responses, $R_{q}$, to 18 questions used Likert-5 scale (from 1—“not at all” through 3—“neutral” to 5—“very much so”), and two were “yes/no” questions.

All questions were tested for differences in responses to the after-game evaluation survey between MP and LP games. The tests were performed with a generalized linear model (GLM): response∼game (Gaussian family for Q1–Q18; binomial for Q19–Q20). The results can be improved with a mixed effects GLM that includes individual random effects and interactions (e.g., with recruitment questionnaire questions reflecting the player’s previous experience with F2P mobile games). One such model is reported in the supporting tables in Data Availability, but here, we proceed with the more conservative GLM results; see Table 2. There were no interactions between the questions and either age, sex, or Bartle’s player type.

The discriminative power of a given question visibly depends on the particular pair of games, with a strong preference in our match-3 pair and little or no preference in the card strategy pair. From among the potentially useful questions, which yielded any significant effect, we selected two groups that were later used for player selection:

(a) Questions directly related to monetization:

Q16 “In general, I would be willing to spend my money playing [game name]”.
Q17 “It is likely that I will play [game name] in the future”.
Q18 “It is likely that I will recommend [game name] to someone”.

(b) Questions related to emotional response to the game:

Q5 “I find playing [game name] interesting”.
Q6 “I think playing [game name] makes you feel joy”.
Q7 “I think playing [game name] is exciting”.
Q8 “I think playing [game name] is fun”.
Q9 “I find playing [game name] relaxes me. I’m resting while playing”.
Q10 “I think the game has a variety of interesting content”.
Q11 “I think I enjoyed playing [game name]”.

### 3.7. Player Index Calculation

The player sensitivity indices were based on the responses to the after-game evaluation survey. To make the indices quantitative, they were defined as weighted sums of differences, $dR_{q}$, between responses regarding an MP and LP game. The differences were rescaled as Likert-5 scores for the base index as $R_{q}\left(PM\right)-R_{q}\left(LM\right)\in \left\{-4,-3,-2,-1,0,1,2,3,4\right\}\to \left\{-1,-1,-0.5,0,0,0,0.5,1,1\right\}∋dR_{q}$ and as Likert-3 scores: $R_{q}\left(PM\right)-R_{q}\left(LM\right)\in \left\{-4,-3,-2,-1,0,1,2,3,4\right\}\to \{-1,-1,$$-1,0,0,0,1,1,1\}∋dR_{q}$. The weights in both indices were chosen to sum to one and result in the range B,S∈[−1,1]. The weights were introduced after a series of consultations with the domain experts from the company based on their experience regarding the opinions of players and the impact of the games. The normalization scheme applied to Likert-5 differences was designed to emphasize strong preferences while reducing the influence of weak or ambiguous ones. In particular, differences of ±3 and ±4 were mapped to ±1, indicating a confident preference; ±2 to ±0.5; and all smaller differences to 0. This piecewise scheme was selected in collaboration with industry experts to reflect a conservative approach—favoring only clear attitudes in the construction of the base index. While linear schemes (e.g., steps of 0.25) offer higher resolution, they risk overemphasizing minor or noisy variations in user responses.

In the experiment, the player’s *base index*, B∈[−1,1], was defined as

$$B=0.25\cdot dR_{16}+0.4\cdot dR_{17}+0.35\cdot dR_{18}$$

and based on questions directly related to monetization.

The player’s *UX sensitivity index*, S∈[−1,1], was defined as follows:

$$\begin{array}{cc} S & =0.15\cdot dR_{5}+0.1375\cdot dR_{6}+0.15\cdot dR_{7}+0.15\cdot dR_{8}\\ \; & +0.1375\cdot dR_{9}+0.1375\cdot dR_{10}+0.1375\cdot dR_{11} \end{array}$$

and it was based on questions related to emotional response to the game (Alternative linear mappings were considered but ultimately not used to preserve robustness against random variation in low-confidence responses).

There are possible external moderators of these indices that are measurable during player recruitment, e.g., time spent on gaming, previous exposure to a given type of games, being an active member of gaming community, or age. These might interact differently depending on the game type. We have not identified any moderator that would reliably predict *B* or *S* in all three studied game types.

### 3.8. Data Selection Based on Player Indices

The 68 players were ranked based on the absolute values of the base index, *B*, value and, separately, the UX sensitivity index, *S*, into several tiers. In further analysis, we only used data from players with |B|,|S|>0.8625 (“highest”) and/or |B|,|S|∈[0.75,0.8625] (“high”). The sample sizes in each game category are provided in Table 3.

## 4. Results

We began the analysis by mining the ATS of entire gameplays and single GAIMs for emotional events in individual players. Then, we designed markers for identifications of differences in the affective spectra characteristic of players who in the survey on gameplay experience expressed the following: willingness to continue the game, recommending the game to friends, and the likelihood of buying IAPs. Lastly, for these players, we performed an analysis of emotion intensity in individual GAIMs.

### 4.1. Detecting GAIM Events via Affective Changes

A sample of the affective spectrum of an individual participant sensitive to the cStr games contrast is presented for the MP game in Figure 4, where we show the five emotions found significant for discriminating this game pair.

Despite overall fluctuations in the time series, we observed noticeable rises and drops in emotion probabilities that consistently align with the onset of specific GAIMs—particularly in the *tutorial* phase—suggesting that the appearance of these modules evokes affective reactions in players. We systematically analyzed rises and drops in these emotion probabilities in relation to GAIM timing to uncover consistent, GAIM-specific affective patterns that may indicate emotionally impactful gameplay elements.

The sample of affective spectra of the other two MP games can be found among the supporting figures in Data Availability.

We then approached the question of whether the ATS can be used in tracing the gameplay dynamics and, specifically, whether it can be used to identify the occurrence of events strongly affecting players within GAIM. Each ATS was segmented according to the appearance of a GAIM. After subtracting the mean from each segment, they were averaged over all GAIM occurrences of all the players. We selected as potentially interesting the changes in time series of a given emotion which were above a standard error away from the mean. Additional criteria for selection were a short onset time (within less than four consecutive frames) and a long-lasting effect (an emotion staying at a new level for at least 1 s) so that the relevant changes could be tracked down in the gameplay recordings. Finally, we visually verified that there were screen changes in the single events that contributed the most to a deviation in a given GAIM.

**Figure 5:**
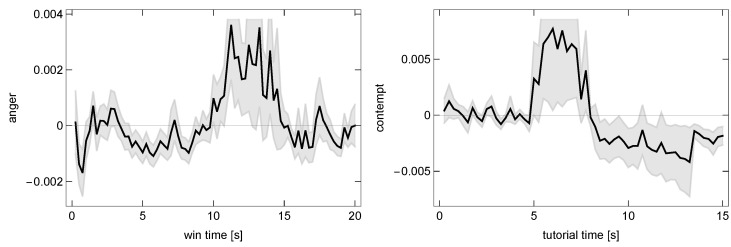
Example fluctuations detected in time courses of GAIMs in m3-MP game. The line is average over players and GAIMoccurrences; the shade is ±1 SD.

In m3-MP, we observed pronounced deviations in the ATS of three GAIMs—tutorial (around 5–8 s after beginning of the GAIM), lost (0–1.5 s), and win (10–15 s)—as shown in Figure 5. After finding individuals that contributed most to these deviations, we found that indeed they were connected to some abrupt screen changes. The won and lost GAIMs comprise sequences of screens (sometimes concurrent with tutorial GAIM) that are designed to dynamically modulate players’ emotional response. It is noteworthy that, within the deviating segments, we were able to identify screens that were not tagged as separate GAIMs but that could induce strong reactions (e.g., one asking for grading player’s satisfaction and another for loading).

In m3-LP, we observed a clear drop in *anger* during the first second of *lost* GAIM. This was, however, not attributable to any screen change but rather to a short emotional reaction. In cStr-MP, we observed similar affective fluctuations, although they were accounted for by a small number of outlying players. No clearly identifiable events in any emotion were found in cStr-LP and, after outlier removals, in the cRPG games.

The varying results serve as a proof of concept that deviations in the ATS can pinpoint specific changes within a GAIM if it has a fairly uniform timing at each occurrence. In more complex designs, where the timing depends on the user interaction, events become desynchronized at the group level but might still be traceable within a single gameplay session. These results also show that affective response can change during a GAIM’s occurrence and that facial recognition at a temporal resolution of the order of a quarter of a second is sufficient to measure it. These affective fluctuations within GAIMs not only reflect moment-to-moment emotional response but also serve as indicators of the impact of key game process events, such as winning, losing, or navigating high-stakes screens. This establishes a meaningful temporal correlation between in-game outcomes and the emotional expression sequence.

### 4.2. Affective Spectra of High-Sensitivity Players

Second, we carried out a hypothesis-driven analysis on the highest B-index players for a given game genre. This was a small group of six players, see Table 3, but due to their high sensitivity, it was best suited to verify the hypothesis that there are identifiable affective spectra characteristic of people who expressed the desire to continue the game, recommending the game to friends, and the likelihood of buying IAPs. Let us stress that calculation of the S and B indices, Section 3.7, and player selection happened prior to the commencement of data analysis.

First, the emotions were assigned signs on the Likert-3 scale (valences), as provided in the first row of Table 4. Then, we compared the affective spectra of MP–LP game pairs according to the following voting scheme: a greater intensity of positive emotions in one of the games than in the other is a vote for the former, while a smaller intensity of negative emotions in one game than the other is a vote for the former. The 0-valence emotions were not considered, nor were the rare cases of votes where the intensity of emotions did not differ significantly. The indicator (which we call *valence voting*, VV) is mathematically a simple scalar product of a nine-dimensional vector of valence and a nine-dimensional vector of differences in the time-averaged intensity of emotions between the two games rescaled to the Likert-3 scale. The resulting VVs for each game were subtracted from each other and finally normalized by the number of non-neutral emotions taking part in the voting.

Given valences as in the first row of the table, we obtained a systematically non-negative VV among the highest-B players. Due to the small sample size, the proper conclusion was to reject the null hypothesis (zero VV). The most adequate formal test was the Wilcoxon matched-pairs signed-ranks test, which yielded a borderline result of p= 0.0100348 at a 0.1 significance level (still too small for such a small sample size [27]). Since the interpretation of automatic emotional recognition and the choice of valences might be disputable and subject to change depending on specific measurement conditions, we further tested other valence vectors, as given in Table 4.

The evaluative indicator in future studies could be defined as the sum of such indicators for all tested players on a pair of games or the same game in two developed versions.

The above analysis is an argument for the adequacy of such a metric. With a larger learning set, one can more optimally match the signs of emotions, for example, change the Likert-3 scale to a more accurate one.

The contribution of each emotion to the final evaluation is weighted through a valence vector, which assigns positive, negative, or neutral values to emotional states. The valence voting (VV) score is computed as a scalar product of this vector with the observed differences in emotion intensity between MP and LP games. Emotions such as happiness or surprise contribute positively, while anger or disgust typically contribute negatively. This ensures that the overall evaluation meaningfully reflects not just emotional intensity but also its affective desirability.

### 4.3. Interactions Between GAIMs and Emotions

**Figure 6:**
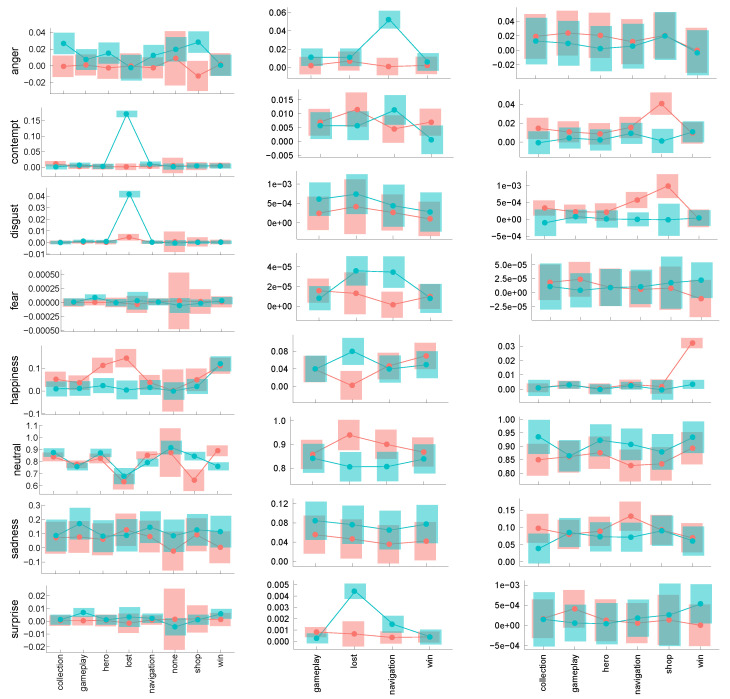
Linear predictions of emotions of high-base-index players by MP (red) and LP (green) games stratified by the available GAIMs. The error bars are lower and upper 95% CIs. (**Left**) Card strategy, (**middle**) match-3, and (**right**) card RPG games. MP games are in red; LP are in green.

The final analysis we present is an exploratory, data-driven one. Statistical analyses were performed using the *lme4* package [28] and the estimated marginal mean package *emmeans* [29]. We used a mixed linear model predicting emotion probability from the game quality (MP/LP) and the GAIM during which the player’s reaction happened, with the participant treated as a random factor. The *tutorial* was treated as a dummy coded variable separate from the other GAIMs due to its overlap with them. The number of modeled and reported GAIMs depended on whether they appeared in both games in a given pair. The approach with a linear mixed model emotion∼game yielded no statistically significant effects. The model including the interaction term game×(GAIM+tutorial) yielded several interesting results. The contrasts between MP and LP games are shown in Figure 6. The contrasts’ significance level was corrected for multiple comparisons using the multivariate t distribution method (mvt) [30]. The exact test results are provided in the supporting tables in Data Availability.

Generally, the results obtained for high-B and high-S are consistent, and the results between game types are different. Interestingly, they verify our assumptions on the choice of valences in the previous section, e.g., anger, disgust, and contempt are mostly markers of card strategy and match-3 LP games, but not of an MP card RPG game; similarly, happiness is a marker of MP games in cStr and cRPG, but this is to the contrary in the match-3 category. The results also show that averaging over the whole gameplay might nullify some effects, and any affective spectrum indicator should rather consider player reactions in separate GAIMs.

To fully utilize the sequence information in the affective time series, we performed segmentation by GAIMs and calculated the average emotional trajectories across players. We applied statistical filtering criteria (e.g., onset latency, duration, standard error thresholds) to detect emotionally salient moments. This allowed us to infer which elements of gameplay consistently elicited strong affective reactions and may therefore contribute to the game’s effectiveness or commercial success.

Since GAIMs often correspond to specific process results (e.g., the player has just won, lost, completed tutorial, or accessed monetization-related content), our analysis of emotional trajectories within GAIMs provides a structured way to link game outcomes to affective dynamics. These statistical results complement the valence voting analysis by showing how specific emotions behave in context. The contrast patterns across GAIMs highlight the importance of individual emotions in shaping user experience and reinforce their role in the overall evaluation framework.

## 5. Discussion

In the present work, we propose a novel approach to testing in an implicit unbiased setting the way players perceive and experience mobile games. By first introducing game success measures based on monetization, we selected more and less profitable game pairs for comparative analysis, and we ran a series of affective experiments on them. In doing so, we validated the main hypothesis of this work that the emotional experience of players can be linked to a game’s potential for success if players can provide reliable emotional responses. Quantitatively, we observed that to robustly use emotions to gauge games performance, we needed to define unique game segments of universal game mechanics function regardless of the particular game example, which we called GAIMs (examples of which are victory screen or main battle screen). These represent the game structure abstraction level and allow for user experience recorded in such segments to be compared among different titles. Second, to actually use human testers’ experience, we discovered the necessity to actually estimate how capable a given player is of experiencing emotions consistently and intensely. To that end, we defined novel sensitivity factors, which could be used to select the most reliable testers. Third, to properly compare emotional or affective signals from different games, we introduced valence factors, attributing positive or negative scores to different emotions reported by the Microsoft Azure system. The goal was to universally map desired and undesired emotions regardless of their superficial perception to humans, e.g., fear may by expected and therefore be a positive reaction (outcome) in certain scenarios, while it may be unwelcome in others, and such valence factors serve as an uniformizer, capturing the game developers intended response of the given game segment. Based on that we defined, indicators differentiating positive and negative examples of games in more profitable and less profitable pairs were explored. Further improvement of the valence factors might be possible by an additional weighting, but a careful and complex assessment is needed first, since emotions seem to interact differently with different GAIMs and game types (or even individual games), as shown in the exploratory analysis in Section 4.3. All these contributions together form a potentially reliable novel framework for gameplay evaluation. Its robustness can be confirmed in practice by game development studios in the process of beta testing or A/B testing of various game mechanics during game development, using wider focus groups by comparing classical assessment methods with our method predictions. Once calibrated (including B/S-index scoring, weighting, and thresholding) or confirmed to work for a given game, the method may then offer significant cost and effort savings for the studio employing the method during subsequent test and design stages.

A potential weakness or reason to dispute the method predictions may occur if the game under study offers little overall emotional response by design or when the study session would make testers tired enough to significantly impair their high emotional sensitivity benefit. The latter is quite easy to control; however, “superficially boring” games pose a greater challenge. Our valence and automated emotion interpretation would be affected in such cases by most emotional spectra collapsing toward the neutral emotion and no smile, yielding no clear interpretation of the GAIMs. We do believe however, that such games constitute a different family of mechanics for different target audiences and perhaps require different kinds of quality assessment metrics. We leave this issue for future study, while strongly emotional games should be tractable by our method.

Another point is that the reported methods are limited to a laboratory setting and entail certain constraints on players (maintaining a pose such that the Azure system can observe the face at all times). On the other hand, our approach and main hypothesis in essence is that engagement is the crucial ingredient leading to success and prolonged user involvement, and emotions are a proxy to gauge its magnitude. We therefore envision a complementary system for measuring engagement in which we use just user interaction with the device in the native setting outside of a laboratory. One can analyze screen taps, the detailed dynamics of interactions with various game elements and menus, the long time scale decay of curiosity reflected by the frequency and intensity of in-game options exploration and use by the player, etc. The online–offline mechanisms of game data mining could lead to a novel broad spectrum of observations in which emotions and physical interactions are used for building a predictive system tracking games performance that is proactively used for game development enhancements in a feedback loop. Beyond that in future work, we plan to incorporate explicit event tagging (e.g., user-initiated purchases, level completion, and reward screens) and apply dynamic time warping or hidden Markov models to further specialize the analysis of emotional sequences in relation to game process structure. The methods introduced in this work, particularly the sensitivity player index and GAIMs, could serve as a basis of such innovations.

## 6. Conclusions

In conclusion, our approach combines temporal structure and emotional valence across gameplay sequences to identify emotionally engaging or discouraging elements, moving beyond surface-level emotion reporting. Our method relies on emotion-level granularity, both through the valence-weighted voting scheme and emotion-specific statistical modeling. This allows us to assign interpretable significance to each emotional channel in the context of evaluating game engagement and quality.

Beta testing and evaluating gameplay in mobile games are challenging due to the much narrower communication channel such games offer as compared to their large platforms counterparts. Even more so, free-to-play games are at the edge of difficulty range due to the success factors of more profitable games that are hard to grasp and the very large churn rate of such games (i.e., the rapid dropping of such games after initial install). To succeed, a free-to-play game must somehow bind players very early on and maintain this bond as long as possible, until enough commitment builds up, so that a player would perceive dropping the game as a loss. Given that mobile free games have become an incredibly competitive industry with high development costs and uncertain monetization, every means to assure publishers’ investment return is of great value. We believe that the method we developed and tested in the present research offers such a contribution to the ever-growing game development industry, and it may serve as a foundation of further, more advanced methods. 

## Figures and Tables

**Table 1:** GAIMs analysed.

Game Type	GAIMs
Match-3	gameplay, navigation, won, lost, (ad)
Card Strategy	gameplay, navigation, won, lost, tutorial, collection, hero, shop, (ad)
Card RPG	gameplay, navigation, won, lost, tutorial, collection, hero, shop, (ad)

**Table 2:** *p*-values of tests for a difference in responses to the after-game evaluation survey between MP and LP games. Statistical significance after Benjamini–Hochberg correction for multiple comparisons is marked in bold at the level * for 0.05 and ** for 0.01.

Question	Match-3	Card RPG	Card Strategy
Q1	0.22	0.025	0.025
Q2	0.052	0.086	0.7
Q3	0.031	0.019	0.47
Q4	**0.009** *	0.11	0.54
Q5	0.11	**0.008** *	0.59
Q6	**0.0004** **	0.03	0.036
Q7	0.22	**0.001** **	0.74
Q8	**0.01** *	0.017	0.14
Q9	**0.0001** **	0.66	**0.002** *
Q10	**0.001** **	0.11	0.95
Q11	**0.002** *	**0.007** *	0.27
Q12	0.12	0.08	0.46
Q13	0.16	0.46	0.45
Q14	0.083	0.41	0.24
Q15	**0.001** **	**0.008** *	0.33
Q16	0.036	0.082	0.66
Q17	**0.005** *	0.077	0.21
Q18	**0.001** **	0.13	0.22
Q19	0.06	**0.009** *	0.7
Q20	0.085	0.33	0.7

**Table 3:** Numbers of participants scoring highest values in terms of indices.

Game Type	Highest B	High B	Highest S	High S
Match-3	5	9	8	5
Card Strategy	1	2	3	2
Card RPG	0	4	1	5

**Table 4:** Summary of valence voting scenarios.

Valences									Match-3					Card Strategy
Anger	Contempt	Disgust	Fear	Happiness	Neutral	Sadness	Surprise	Smile	id07	id12	id41	id55	id56	id47
−1	−1	−1	1	1	0	−1	1	1	0.13	0.25	0.75	0.50	0	0
−1	−1	−1	0	1	0	−1	1	1	0.14	0.43	0.71	0.71	0.14	−0.14
−1	0	0	0	1	1	−1	0	1	−0.20	1.0	1.0	1.0	−0.20	0.20
−1	1	−1	1	1	0	−1	1	1	−0.13	0.50	1.0	0.75	0.25	−0.25
−1	−1	−1	−1	1	0	−1	1	1	0.13	0.50	0.50	0.75	−0.25	0.25

## Data Availability

The questionnaires, facial recognition time series, supporting figures, and tables with statistical test results are available at https://osf.io/vxwa9/ (accessed on 14 February 2025).

## References

[1] Yannakakis G.N., Togelius J. (2018). Artificial Intelligence and Games.

[2] Drachen A., Thurau C., Togelius J., Yannakakis G., Bauckhage C. (2013). Game Data Mining.

[3] El-Nasr M.S., Canossa A., Nguyen T.H.D., Drachen A. (2021). Game Data Science.

[4] Picard R.W. (1997). Affective Computing.

[5] Hudlicka E. Affective Computing for Game Design. Proceedings of the 4th International North American Conference on Intelligent Games and Simulation (GAMEON-NA).

[6] Yannakakis G.N., Paiva A. (2014). Emotion in games. Handbook on Affective Computing.

[7] Brühlmann F., Mekler E.D., Drachen A., Mirza-Babaei P., Nacke L. (2018). Surveys in Games User Research. Games User Research.

[8] Bromley S., Drachen A., Mirza-Babaei P., Nacke L. (2018). Interviewing players. Games User Research.

[9] Nalepa G.J., Kutt K., Giżycka B., Jemioło P., Bobek S. (2019). Analysis and Use of the Emotional Context with Wearable Devices for Games and Intelligent Assistants. Sensors.

[10] Bandara D., Song S., Hirshfield L., Velipasalar S. A more complete picture of emotion using electrocardiogram and electrodermal activity to complement cognitive data. Proceedings of the International Conference on Augmented Cognition.

[11] Zhang X., Xu C., Xue W., Hu J., He Y., Gao M. (2018). Emotion Recognition Based on Multichannel Physiological Signals with Comprehensive Nonlinear Processing. Sensors.

[12] Ringeval F., Eyben F., Kroupi E., Yuce A., Thiran J.P., Ebrahimi T., Lalanne D., Schuller B. (2015). Prediction of asynchronous dimensional emotion ratings from audiovisual and physiological data. Pattern Recognit. Lett..

[13] Brady K., Gwon Y., Khorrami P., Godoy E., Campbell W., Dagli C., Huang T.S. Multi-modal audio, video and physiological sensor learning for continuous emotion prediction. Proceedings of the 6th International Workshop on Audio/Visual Emotion Challenge.

[14] Yin Y., Nabian M., Ostadabbas S. (2018). Facial expression and peripheral physiology fusion to decode individualized affective experience. arXiv.

[15] Valstar M., Gratch J., Schuller B., Ringeval F., Lalanne D., Torres Torres M., Scherer S., Stratou G., Cowie R., Pantic M. Depression, mood, and emotion recognition workshop and challenge. Proceedings of the 6th International Workshop on Audio/Visual Emotion Challenge.

[16] AlZoubi O., Fossati D., D’Mello S., Calvo R.A. (2019). Affect detection from non-stationary physiological data using ensemble classifiers. Sensors.

[17] Tzirakis P., Trigeorgis G., Nicolaou M.A., Schuller B.W., Zafeiriou S. (2017). End-to-end multimodal emotion recognition using deep neural networks. IEEE J. Sel. Top. Signal Process..

[18] Basu S., Jana N., Bag A., Mahadevappa M., Mukherjee J., Kumar S., Guha R. Emotion recognition based on physiological signals using valence-arousal model. Proceedings of the 2015 Third International Conference on Image Information Processing (ICIIP).

[19] Granato M. (2019). Emotions Recognition in Video Game Players Using Physiological Information. Doctoral Thesis.

[20] Kutt K., Drążyk D., Bobek S., Nalepa G.J. (2021). Personality-Based Affective Adaptation Methods for Intelligent Systems. Sensors.

[21] Ekman P., Friesen W. (1976). Pictures of Facial Affect.

[22] Nacke L.E., Drachen A., Mirza-Babaei P., Nacke L. (2018). Introduction to biometric measures for games user research. Games User Research.

[23] Carcagnì P., Coco M., Leo M., Distante C. (2015). Facial expression recognition and histograms of oriented gradients: A comprehensive study. SpringerPlus.

[24] Li S., Deng W. (2020). Deep Facial Expression Recognition: A Survey. IEEE Trans. Affect. Comput..

[25] Küntzler T., Höfling T.T.A., Alpers G.W. (2021). Automatic Facial Expression Recognition in Standardized and Non-standardized Emotional Expressions. Front. Psychol..

[26] Richard A. (1996). Bartle: Players Who Suit MUDs. https://mud.co.uk/richard/hcds.htm.

[27] Dhand N., Khatkar M. (2014). Statulator: An Online Statistical Calculator. Sample Size Calculator for Comparing Two Paired Means. http://statulator.com/SampleSize/ss2PM.html.

[28] Bates D., Mächler M., Bolker B., Walker S. (2015). Fitting Linear Mixed-Effects Models Using lme4. J. Stat. Softw..

[29] Lenth R.V. (2021). emmeans: Estimated Marginal Means, aka Least-Squares Means. https://CRAN.R-project.org/package=emmeans.

[30] Kotz S., Nadarajah S. (2004). Multivariate T-Distributions and Their Applications.

